# Retraction: CircRNA circPDSS1 promotes bladder cancer by downregulating miR-16

**DOI:** 10.1042/BSR-2019-1961_RET

**Published:** 2023-03-02

**Keywords:** circPDSS1, miR-16, urothelial bladder cancer

This Retraction follows an Expression of Concern relating to this article previously published by Portland Press.

This article is being retracted from *Bioscience Reports* at the request of the Editor-in-Chief and the Editorial Board following receipt of a notification from a reader, alerting the Editorial Board to repeated images in Figure 5.

The Editorial Office was also contacted by the listed corresponding author, Derong Ma, via an email address that differed to the one included with the submission. Derong Ma confirmed that they were unaware of the submission and publication of the article, and that they did not conduct any part of this research, hence should not have been listed as an author on this paper.

The corresponding author of the paper has been contacted via the original email address in the published paper to provide a response to the Editorial Office concerns surrounding the authorship of the paper but has not responded.

In light of the concerns raised regarding the Western blots and the authorship of the paper, the Editorial Board stands by the decision to retract the paper.

